# Identified mental disorders in older adults in primary care: A cross-sectional database study

**DOI:** 10.1080/13814788.2017.1402884

**Published:** 2018-01-22

**Authors:** Geoff McCombe, Frank Fogarty, Davina Swan, Ailish Hannigan, Gerard M. Fealy, Lorraine Kyne, David Meagher, Walter Cullen

**Affiliations:** ^a^ UCD School of Medicine, University College Dublin Dublin Ireland; ^b^ Institute of Psychiatry, King’s College London London UK; ^c^ Graduate-Entry Medical School, University of Limerick Limerick Ireland; ^d^ UCD School of Nursing, Midwifery and Health Systems, University College Dublin Dublin Ireland

**Keywords:** Mental disorders, electronic medical records, general practice, primary healthcare, prevalence, treatment

## Abstract

**Introduction:** Identifying and managing mental disorders among older adults is an important challenge for primary care in Europe. Electronic medical records (EMRs) offer considerable potential in this regard, although there is a paucity of data on their use for this purpose.

**Objectives:** To examine the prevalence/treatment of identified mental disorders among older adults (over 55 years) by using data derived from EMRs in general practice.

**Methods:** We utilized data from a cross-sectional study of mental disorders in primary care, which identified patients with mental disorders based on diagnostic coding and prescribed medicines. We collected anonymized data from 35 practices nationally from June 2014 to March 2015, and secondary analysis of this dataset examined the prevalence of mental disorders in adults aged over 55 years.

**Results:** 74,261 patients aged over 55 years were identified, of whom 14,143 had a mental health disorder (prevalence rate of 19.1%). There was considerable variation between practices (range: 3.7–38.9%), with a median prevalence of 23.1%. Prevalence increased with age, from 14.8% at 55–59 years to 28.9% at 80–84 years. Most common disorders were depression (17.1%), panic/anxiety (11.3%), cognitive (5.6%), alcohol (3.8%) and substance use (3.8%).

**Conclusions:** Examining mental disorders among older adults using data derived from EMRs is feasible. Mental disorders are common among older adults attending primary care and this study demonstrates the utility of electronic medical records in epidemiological studies of large populations in primary care.

KEY MESSAGES

- The mental health finder tool is a feasible and promising methodology for large-scale data collection on mental health disorders among older adults in primary care.
- Given the magnitude of the observed variability, further study is needed to better understand the causes of variable practice-level diagnosis and prescribing.

## Introduction

In Europe, the number of older people has increased significantly since 2000 and it is projected to continue to do so until 2050. More information on morbidity rates amongst older people is therefore required to plan for healthcare services [1]. As people are living longer, the disease burden changes from communicable diseases to chronic conditions, including mental disorders. The most common mental disorders in older people include depression, anxiety, and substance abuse [2]. Mental disorders are associated with increased healthcare costs, mortality and suicide, along with interference with daily living, and a reduction in quality of life [3].

Mental health policy in Ireland is guided by *A Vision for Change*, which advocates a holistic, multidisciplinary approach to addressing mental health, with an emphasis on treating mental disorders in the community [4]. In relation to mental health in older adults, the policy points to the importance of primary care services. While primary care professionals may be well placed to address the needs of people with mental health problems, little contemporaneous data exists on the prevalence and management of mental health conditions, especially among older patients attending primary care [5].

Amongst older adults, depression is the commonest mental disorder [6]. The prevalence of mental disorders amongst older adults in Sweden was 33%, with dementia (16%), depression (12%) and anxiety (11%) the most common conditions [3]. A meta-analysis of mental disorders in older adults found the lifetime prevalence of affective disorders was 16.52%, anxiety disorders 2.63%, substance abuse disorders 11.71% and psychosis 4.7% [1]. In Ireland, the ‘Longitudinal Study on Ageing (TILDA)’ found that 10% of participants self-reported clinically significant depression, while 18% self-reported sub-clinical depression; of these, only 5% had been diagnosed with depression [7].

Electronic medical records (EMRs) are becoming more commonly used to analyse primary care morbidity data [8–10]. A systematic review aimed at addressing some of the challenges of using this method to analyse big data identified all case definitions for a set of chronic conditions that have been tested and validated in primary care EMR and EMR-linked data [11]. The rich information contained in EMRs in primary care offers considerable potential for research purposes. In the area of mental disorders, this may overcome many of the challenges in recruiting and selecting participants for research [12], while anonymizing data at the source of collection protects the privacy of patients [13]. Furthermore, database-enabled research has recently been identified as a priority area for mental health research in Europe [14]. However, there is a paucity of research using EMRs to measure the prevalence of mental disorders in older people.

Swan et al. developed a software tool for a commonly used general practice management system to identify patients with common mental disorders [15]. By way of a secondary analysis of this dataset, we aimed to address the lack of population data on the prevalence of identified mental health disorders among older adults by focusing on the following study questions.

- What proportion of patients aged 55 and older is identified with a mental health condition?
- How does cumulative lifetime prevalence increase as patients become older?
- What are the most common mental health conditions in this population?
- What practice factors are associated with a higher prevalence of mental health conditions in the older adult?

## Methods

### General study design

*Recruitment of practices.* We sought expressions of interest from practices affiliated with the host institution that used the same practice management/EMR system for at least the previous two years. Practices were recruited to the study if, following a further discussion with the research team about the study, they were happy to facilitate data collection.

GPs record any relevant mental health information and prescribed medication into the patient’s EMR during a consultation. A ‘mental health finder’ software tool, embedded within a commonly used practice management systems (Socrates™) was developed to search a practice’s EMRs to find those patients who had been assigned a predetermined mental health diagnostic code or who had been prescribed a psychotropic medication at any point in time.

A 30-minute training session was agreed as being sufficient to train each GP in using the ‘finder’. GPs were also given guidance notes on how to use the ‘finder’ and the contact details of the researcher should they encounter any problems.

### Data collection

We computerized remote data extraction on common mental and substance use disorders at 35 general practices nationally, serving a population of over 200,000 patients between June 2014 and March 2015. The ‘finder’ focussed on mental health problems most commonly encountered in primary care, i.e., depression, panic/anxiety, somatoform disorders, eating disorders and alcohol disorders, substance use disorders, cognitive disorders, and other (unspecified) psychological disorders [15]. We reviewed the International Classification of Primary Care, second edition (ICPC2) codes [16] disease and symptom/complaint codes for coding conditions/problems, and the International Classification of Diseases,10th Revision (ICD-10) codes [17], and we included those relating to the diagnosis or classification of the mental health conditions. Regarding prescriptions, medications within the ‘Anatomical and Therapeutic Chemical (ATC)’ classes were incorporated in the finder; these were: antipsychotics (N05A), anxiolytics (N05B), hypnotics and sedatives (N05C), antidepressants (N06A), psychostimulants, agents used for ADHD and nootropics (N06B), psycholeptics and psychoanaleptics in combination (N06C), anti-dementia drugs (N06D), and methadone (N02AC52). The development of this tool and its functionality has been described in detail elsewhere [15]. As well as data on patients with mental disorders, practice level data, such as denominator population, training and access to mental health services, were also collected. All data collected were anonymized

The variables used in Tables 1–4 display the prevalence of the most common mental disorders and prescribed medications outlined above.

**Table 1.** Prevalence of common mental disorders amongst participating practices.

Disorder	Range across participating practices %	Median prevalence across participating practices %	Mean prevalence across participating practices %	SD
Depression	0.3–37.9	13.3	15.1	11.17
Panic/anxiety	0.2–39.6	4.1	7.63	9.30
Cognitive	0.3–26.4	2.9	4.95	5.51
Alcohol	0.7–12.1	3.55	4.59	3.77
Substance misuse	0.1–13.7	0.65	3.16%	3.97

Depression, coded diagnosis of depression in the EMR; Panic/anxiety, coded diagnosis of a panic/anxiety disorder in the EMR; Cognitive, coded diagnosis of a cognitive disorder in the EMR; Alcohol, coded diagnosis of an alcohol disorder in the EMR; Substance misuse, coded diagnosis of a substance use disorder in the EMR. SD: standard deviation.

**Table 2.** Influence of gender on morbidity in participating practices.

Disorder	% Females	% Males	Chi-square
Depression	17.9	15.8	0.001
Panic/anxiety	12.3	9.7	<0.001
Alcohol	1.8	7.4	<0.001
Substance	3.5	4.4	0.004
Any disorder	33.5	35.6	0.011
Drug class			
Anxiolytics	29.8	23.9	<0.001
Hypnotics/sedatives	41.2	33.4	<0.001
Antidepressants	47.5	42.2	<0.001
Any drug	88.6	83.4	<0.001

Depression, coded diagnosis of depression in the EMR; Panic/anxiety, coded diagnosis of a panic/anxiety disorder in the EMR; Alcohol, coded diagnosis of an alcohol disorder in the EMR; Substance, coded diagnosis of a substance use disorder in the EMR; Any disorder, coded diagnosis of any mental health or substance use disorder in the EMR; Drug class, medication prescribed for a mental health disorder in the EMR.

**Table 3.** Influence of General Medical Scheme (GMS) status on morbidity in participating practices.

Disorder	% Non-GMS	% GMS	Chi-square
Panic/anxiety	13.8	10.5	<0.001
Substance	2.9	4.1	0.001
Cognitive	4.0	6.2	<0.001
Other disorder	0.5	0.9	0.024
Any disorder	36.4	33.4	0.001
Drug class			
Antipsychotic	9.4	18.6	<0.001
Anxiolytics	25.9	28.3	0.006
Hypnotics/sedatives	30.4	41.2	<0.001
Antidepressants	38.4	48.1	<0.001
Anti-dementia	2.1	7.8	<0.001
Any drug	77.3	90.1	<0.001

Panic/anxiety, coded diagnosis of a panic/anxiety disorder in the EMR; Substance, coded diagnosis of a substance use disorder in the EMR; Cognitive, coded diagnosis of a cognitive disorder in the EMR; Other disorder, coded diagnosis of other mental health disorder in the EMR; Any disorder, coded diagnosis of any mental health or substance use disorder in the EMR; Drug class, medication prescribed for a mental health disorder in the EMR; Any drug, any prescribed mental health medication in the EMR.

**Table 4.** Influence of practice type on morbidity in participating practices.

Disorder	% Rural	% Urban	Chi-square
Depression	15.1	17.6	0.001
Panic/anxiety	5.4	12.9	<0.001
Alcohol	2.9	4	0.003
Substance misuse	0.9	4.6	<0.001
Cognitive	2.9	6.3	<0.001
Other	0.1	0.9	<0.001
Any disorder	24.8	36.7	<0.001
Drug class			
Antidepressants	49.6	44.5	<0.001
Psychostimulants	0.1	0.7	<0.001
Any drug	89.6	85.9	<0.001

Depression = coded diagnosis of depression in the EMR; Panic/anxiety, coded diagnosis of a panic/anxiety disorder in the EMR; Alcohol, coded diagnosis of an alcohol disorder in the EMR; Substance misuse, coded diagnosis of a substance use disorder in the EMR; Cognitive, coded diagnosis of a cognitive disorder in the EMR; Other disorder, coded diagnosis of other mental health disorder in the EMR; Any disorder, coded diagnosis of any mental health or substance use disorder in the EMR; Drug class, medication prescribed for a mental health disorder in the EMR: Any drug, any prescribed mental health medication in the EMR.

### Data analysis

Data handling was supported using Microsoft Excel and SPSS Version 20.0 (SPSS Inc., Chicago IL). We used frequency distributions, measures of central tendency and measures of variability to describe the main variables of interest and chi-square analysis was used to identify group differences on variables of interest. A multivariate analysis was carried out to examine the relationship between being diagnosed with ‘any disorder’ and various potential predictors (‘type of area,’ gender, and ‘GMS status’) (Table 5).

**Table 5.** Summary of multiple regression analysis for variables affecting being diagnosed with a mental health disorder in participating practices.

Variable	B	SE B	β	*P*
Type of area, urban/rural	0.195	0.007	0.149	0
Gender, male/female	0.024	0.006	0.024	0
Status (GMS^a^ etc.)	0.015	0.003	0.032	0

B: the unstandardized beta; SE B: the standard error for the unstandardized beta; β: the standardized beta.

^a^ GMS/non GMS. Under Ireland’s General Medical Scheme (GMS) patients on low income are entitled to free GP care.

### Ethics

The Research Ethics Committee of the Irish College of General Practitioners approved the study.

## Results

### Study population

Thirty-five practices participated in the study, of whom 30 provided information on the treatment of mental use disorders in their practice, 24 (80%) had completed specialist GP training, seven (23%) had completed a diploma or certificate in mental health/substance use, 26 (87%) participated in continuing medical education (CME), and 22 (73.3%) had completed a course on substance abuse. Twenty-two (73%) reported their postgraduate training prepared them adequately to deal with adult mental health, while 11 (37%) reported it had prepared them to adequately deal with substance use. Twenty-seven (90%) had counselling services available for patients entitled to free GP care under Ireland’s General Medical Scheme (hereafter GMS-eligible) and nine (30%) provided a counselling service at the practice. For GMS-eligible patients, three (15%) reported the waiting time for counselling was one-to-three weeks, 11 (55%) reported the timeframe was one-to-three months and six (30%) reported it was longer than three months.

### Prevalence of identified mental disorders

**Figure 1.**
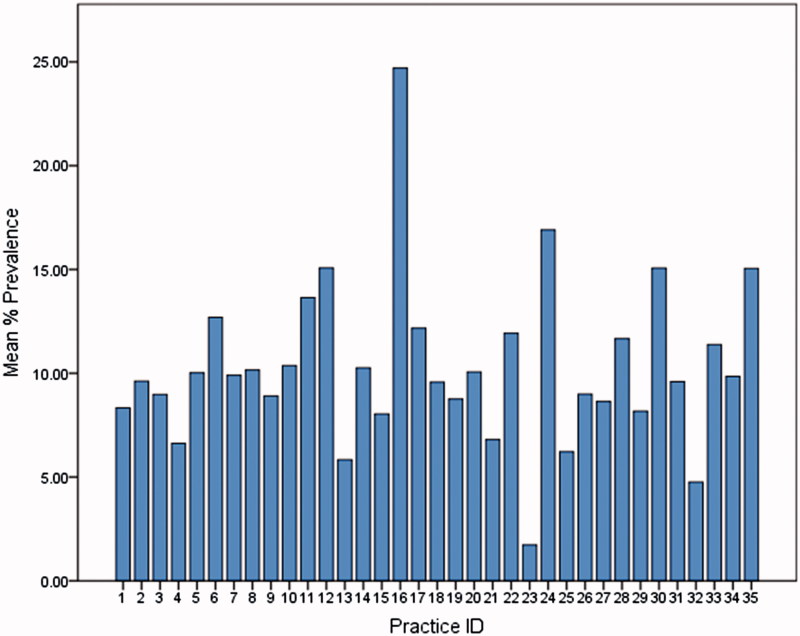
Variation in prevalence in identified mental disorders between practices.

**Figure 2.**
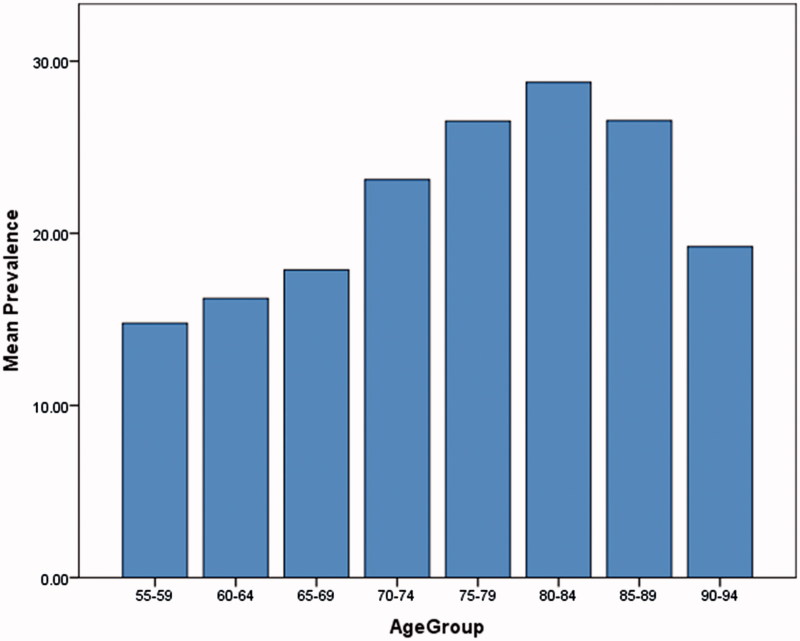
Variation in prevalence between age groups.

Of 74,261 patients aged 55 years and older, 14,143 were identified as having a mental disorder, representing an overall prevalence of 19.1% (95% confidence interval [CI] ± 0.3%). There was considerable variation between practices in prevalence (Figure 1) and the median prevalence was 23.1% (range: 3.7–38.9%). The prevalence increased with age, from 14.8% among patients aged 55–59 years to 28.8% among patients aged 80–84 years (Figure 2).

Of those identified, 9070 (64.1%) were female and 9982 (70.6%) were GMS-eligible. The most commonly prescribed drugs were antidepressants (45.6%, *n* = 6445), followed by hypnotics/sedatives (38.4%, *n* = 5429), anxiolytics (27.7%, *n* = 3917), and antipsychotics (16.2%, *n* = 2290). The most common disorder identified was depression (15.1%, *n* = 2421). There was considerable variation between practices in each condition’s identified prevalence (Table 1).

### Factors associated with mental disorders

There was considerable variation in morbidity between genders (Table 2). Men were significantly more likely to be diagnosed with ‘any disorder’ (odds ratio [OR]: 1.1; 95%CI: 10–1.2), an ‘alcohol disorder’ (OR: 4.4; 95%CI: 3.7–5.4) or ‘substance use disorder’ (OR: 1.3; 95%CI: 1.1–1.5).

There were also considerable differences in morbidity between GMS-eligible and non-eligible patients (Tables 3 and 4). GMS-eligible patients were significantly more likely to be diagnosed with ‘substance use disorder’ (OR: 1.4; 95%CI: 1.1–1.7), ‘cognitive disorder’ (OR: 1.6; 95%CI: 1.3–1.9) ‘or other disorders’ (OR: 1.8; 95%CI: 1.1–3.0). GMS-eligible patients were also more likely to be prescribed ‘any drug’ (OR: 2.7; 95%CI: 2.4–3.0).

Patients attending ‘mostly urban’ practices were significantly more likely to be diagnosed with ‘any disorder’ (OR: 1.757; 95%CI: 1.601–1.927), ‘depression’ (OR: 1.205; 95%CI: 1.077–1.349), ‘panic/anxiety’ (OR: 2.566; 95%CI: 2.166–3.040), ‘alcohol misuse’ (OR: 1.122; 95%CI: 1.805), ‘substance misuse’ (OR: 5.092; 95%CI: 3.450–7.514), ‘cognitive’ (OR: 2.234; 95%CI: 1.777–2.809) or ‘other disorders’ (OR: 9.199; 95%CI: 2.918–29.001) and to be prescribed ‘psychostimulants’ (OR: 10.001; 95%CI: 2.455–40.734).

Multivariate analysis showed that ‘type of area,’ gender, and ‘GMS status’ significantly predicted being diagnosed with ‘any disorder’ (Table 5).

## Discussion

### Main findings

This first large population study to examine mental disorders in older adults in Ireland highlights that these disorders are common among older adults attending general practice. The overall prevalence was 19.05% and the median prevalence 23.05%. The most common mental disorders identified were depression (17.1%), panic/anxiety disorder (11.3%) and cognitive disorder (5.6%), with antidepressants (45.6%), hypnotics/sedatives (38.4%) and anxiolytics (27.7%) the most commonly prescribed drugs. While men were significantly more likely to be diagnosed with alcohol use disorder or substance use disorder, women were more likely to be diagnosed with panic/anxiety, depression and to be prescribed antidepressants, anxiolytics or hypnotics/sedatives. GMS-eligible patients were more likely to be prescribed medication and less likely to be diagnosed with panic/anxiety disorders. Those attending ‘mostly urban’ practices were more likely to be diagnosed with a disorder, while those attending mostly rural practices were more likely to be prescribed antidepressants or any drug.

### Relation to existing literature

Internationally, much of the research to date has focussed on measuring prevalence of mental disorders in primary care through face-to-face evaluations using structured clinical interviews [18], PRIME-MD questionnaire [19], and questionnaires [20]

There is little consensus on the overall prevalence of mental disorders in older adults. Our findings are consistent with previous studies that reported depression as the most common disorder [6]. Research comparing the prevalence of common mental disorders in general practice attendees across Europe showed high variation between countries [21]. Compared to the King et al., study, our data for depression in females was similar to Portugal (17.9 compared to 17.8 in Portugal) and Spain (18.4) but much higher than other countries. For depression in males, our data (15.8) is higher than other European countries (4.4–12.7). Compared to other European research studies, our findings are comparable to Sweden for anxiety, higher for depression but lower for dementia (5.6% cognitive disorders compared to 16% dementia in Sweden) [10], and higher for depression compared to Wales (17.1 compared to 9.3) [22] The rate of diagnosed depression is considerably higher in our study population (17.1%) than that reported in Ireland’s National Longitudinal Study on Ageing (5%) [6]. Previous attempts to estimate the prevalence of alcohol misuse in older people have proven difficult for a variety of reasons, including a tendency on the part of study respondents to under-report usage [23]. Research from the UK and the USA showed that older men were 2.77 times and 3.72 times, respectively, more likely than older women to drink in excess of the recommended limit [23,24]. Our study shows that older men in Ireland are 4.446 times more likely than older women to be diagnosed with an alcohol disorder by their GP (95%CI: 3.682–5.368).

Much of the literature points to a relationship between socio-economic status and mental disorders. Our closest measure for this phenomenon is GMS status, specifically whether or not the patient has a medical card or doctor visit card (i.e., is GMS-eligible). In Ireland, the GMS scheme provides payment for GP services for patients with lower incomes, although those aged 70 years and older are more likely to be GMS-eligible than younger patients. This study shows that GMS-eligible patients are more likely to be diagnosed with substance, cognitive or other disorders or to be prescribed any of the major classes of drugs. Non-GMS patients were more likely to be diagnosed with panic or anxiety disorders.

### Strengths and limitations

This study was the largest retrospective study to date of older adults with mental disorders attending general practices in Ireland. The 35 GP practices were representative of general practices across Ireland, including those in urban and rural areas, cities and towns, with GMS and private patients, as well as GP practices of different sizes and with varying levels of resources and capabilities in relation to within-practice mental health services.

While participating practices were reflective of general practice in Ireland, we acknowledge some sources of potential bias resulting from participating practices volunteering to participate in the study and from their using the same EMR system.

The large variability in the results is consistent with the extant research of mental health disorders within primary care [25–27]. Reasons for the large variability in prevalence between practices includes the fact that urban practices are likely to have greater access to a wider range of mental health services and this may result in these practices having systematic screening programmes to identify behavioural or emotional disorders. Also the different care experience received by patients, depending on GP training, experience, and self-efficacy in diagnosing and treating particular mental health conditions is a further possible explanation for variability. Prevalence figures can also vary due to differences in sensitivity and specificity in the range of screening instruments used in diagnosing mental health disorders. While some screening instruments capture specific mental health diagnoses, for example CIDI [28], others capture symptoms through self-administered questionnaires, like the PHQ [29]. Also, some tools identify caseness (e.g., GHQ), while others generate actual specific diagnoses (e.g., PRIME-MD); the sensitivity of the latter is usually lower and this is obviously relevant to an epidemiological report. Finally, inconsistent recording of patient information into EMRs by GPs and the use of different software systems may also increase variability of results [30,31]. The capacity of the mental health finder tool to efficiently extract and analyse large-scale mental health data should be used to highlight to GPs the necessity to code and record patients’ mental health activity consistently and accurately so as to optimize the potential of the ‘finder tool’ to inform service planning and quality improvement.

### Implications for education, clinical practice, policy and future research

Mental disorders are common among older adults attending general practices and therefore their optimum identification and treatment are a priority. Demographic and contextual factors are associated with patterns of morbidity and this has implications for service delivery. Although further research that utilizes patient interviews will be important to more fully examine mental health disorders and their treatment in primary care, this study suggests that among older populations, EMR derived data on common mental and substance use disorders provides data on lifetime prevalence that is comparable with data derived from approaches that are more traditional.

## Conclusions

Examining mental disorders among older adults using data derived from EMRs is feasible. However, given the magnitude of the observed variability, further study is needed to better understand the causes of variable practice-level diagnosis and prescribing, and the implication for health outcomes in older adults.
